# Diagnosis and Management of Gastroesophageal Reflux Disease

**DOI:** 10.1155/2013/709620

**Published:** 2013-11-06

**Authors:** Ping-I Hsu, Nayoung Kim, Khean Lee Goh, Deng-Chyang Wu

**Affiliations:** ^1^Division of Gastroenterology, Department of Internal Medicine, Kaohsiung Veterans General Hospital, National Yang-Ming University, Kaohsiung 813, Taiwan; ^2^Division of Gastroenterology, Department of Internal Medicine, Seoul National University Bundang Hospital, Seoul National University, Republic of Korea; ^3^Department of Medicine, University of Malaysia, Kuala Lumpur, Malaysia; ^4^Division of Gastroenterology, Department of Internal Medicine, Kaohsiung Medical University Hospital, Kaohsiung 807, Taiwan

Gastroesophageal reflux disease (GERD) is one of the most common disorders in medical practice. It is the most common gastrointestinal diagnosis recorded during visits to outpatient clinics in the United States. Apart from the economic burden of the disease and its impact on quality of life, GERD is the most common predisposing factor for esophageal adenocarcinoma [1].

Recently, many important issues have emerged regarding the classification, pathogenesis, natural history, and treatment of GERD. Although use of proton-pump inhibitor (PPI) is the treatment of choice for GERD, approximately, one-third of patients with GERD fail to response symptomatically to a standard-dose proton-pump inhibitor (PPI), either partially or completely [2]. Additionally, most GERD patients need long-term treatment for frequent relapses after discontinuing acid inhibition therapy. This has led to great interest in new endoscopic therapies for the treatment of this disease. With regard to the diagnosis of GERD, patients with refractory reflux symptoms and normal upper endoscopy are more difficult to diagnose and treat. Combined 24-hour pH and impedance monitoring allows classifying the patients as having true nonerosive reflux disease (NERD), hypersensitive esophagus, or functional heartburn and is helpful for further management of the patients [3].

The main focus of this special issue is on recent advances in the treatment of erosive esophagitis, NERD and Barrett's esophagus. In addition, the emerging diagnostic methods, pharmacological treatments, and endoscopic therapies for GERD are also discussed.

The paper entitled “*The frequencies of gastroesophageal and extragastroesophageal symptoms in patients with mild erosive esophagitis, severe erosive esophagitis, and Barrett's esophagus*, in Taiwan” is the first work simultaneously assessing the differences in reflux symptom profiles among the three different categories of GERD. The data showed that the frequencies of some esophageal and extraesophageal symptoms in patients with Los Angeles grade A/B erosive esophagitis were higher than those in patients with Los Angeles grade C/D erosive esophagitis and Barrett's esophagus. 

In the paper entitled “*Current pharmacological management of gastroesophageal reflux disease*,” Y.-K. Wang et al. present the current and developing therapeutic agents for GERD treatment. The efficacies of PPIs and potassium-competitive acid blocker in GERD therapy are well reviewed. Additionally, the article summarizes the development of novel therapeutic agents focusing on the underlying mechanisms of GERD.

In the paper entitled “*Pharmacological therapy of gastroesophageal reflux in preterm infants*,” L. Corvaglia et al. review the pathogenesis, presentation, diagnosis, and treatment of gastroesophageal reflux in preterm infants. A stepwise approach is advisable for the treatment of gastroesophageal reflux in preterm infants, firstly, promoting nonpharmacological interventions and secondly, limiting drugs to selected infants unresponsive to the conservative measures or who are suffering from severe gastroesophageal reflux with clinical complications.

In the paper entitled “*Stretta radiofrequency treatment for GERD: a safe and effective modality*,” M. Franciosa et al. focus on the safety, efficacy, and durability of the Stretta radiofrequency treatment for GERD therapy. The novel endoscopic treatment reduces esophageal acid exposure, decreases the frequency of transient lower esophageal relaxation, decreases medication use and improves quality of life in GERD patients. 

In the paper entitled “*Duodenal tube feeding: an alternative approach for effectively promoting weight gain in children with gastroesophageal reflux and congenital heart disease*,” S. Kuwata et al. showed that duodenal tube feeding improves the weight gain of infants with gastroesophageal reflux who need treatment for congenital-heart-disease-associated heart failure.

In the paper entitled “*Changes in ghrelin-related factors in gastroesophageal reflux disease in rats*,” M. Nahata et al. examined gastrointestinal hormone profiles and functional changes in rats with GERD. The results suggest that aberrantly increased secretion of peripheral ghrelin and decreased ghrelin responsiveness may occur in GERD rats.

In the paper entitled “*Surgical management of pediatric gastroesophageal reflux disease*,” H. T. Jackson and T. D. Kane review the clinical presentation of GERD in pediatric population and discuss the options for surgical management and outcome in these patients.

In the paper entitled “*Current advances in the diagnosis and treatment of nonerosive reflux disease*,” C. L. Chen and P. I. Hsu, review the literature about the pathogenesis, natural history, diagnosis and treatment of NERD. The authors suggest that a combination of 24-hour esophageal impedance and pH monitoring is indicated to differentiate acid-reflux-related NERD, weakly acid reflux-related NERD (hypersensitive esophagus), nonacid-reflux-related NERD, and functional heartburn in patients with poor response to appropriate PPI treatment.

In the paper entitled “*Antireflux endoluminal therapies: past and present*,” K. C. Yew et al. and S.-K. Chuah review, highlight, and discuss three commonly employed antireflux endoluminal procedures: fundoplication or suturing techniques (EndoCinch, NDO, EsophyX), intramural injection or implant techniques (enhancing LES volume and/or strengthening compliance of the LES-EnteryX, Gatekeeper), and radiofrequency ablation of lower esophageal sphincter and cardia (the Stretta system). 

*Ping-I Hsu* *Ping-I Hsu* *Nayoung Kim* *Nayoung Kim* *Khean Lee Goh* *Khean Lee Goh* *Deng-Chyang Wu* *Deng-Chyang Wu*
